# PERSONALITY, SOCIAL ISOLATION, AND LONELINESS: CROSS-SECTIONAL DATA FROM THE CANADIAN LONGITUDINAL STUDY ON AGING

**DOI:** 10.1093/geroni/igad104.2787

**Published:** 2023-12-21

**Authors:** Jennifer Bethell, Melissa Andrew, Debra Morgan, Megan O’Connell, Natalie A Phillips, Walter Wittich, Katherine McGilton

**Affiliations:** Toronto Rehabilitation Institute - University Health Network, Toronto, Ontario, Canada; Dalhousie University, Halifax, Nova Scotia, Canada; University of Saskatchewan, Saskatoon, Saskatchewan, Canada; University of Saskatchewan, Saskatoon, Saskatchewan, Canada; Concordia University, Montreal, Quebec, Canada; Université de Montréal, Montreal, Quebec, Canada; Toronto Rehabilitation Institute - University Health Network, Toronto, Ontario, Canada

## Abstract

Good social connection is associated with better health. However, the relationships between distinct aspects of objective and subjective social connection, and how personality traits may impact these relationships, are not well-understood. For example, in predicting loneliness, personality could moderate the impact of objective social connection by influencing the way social relationships are evaluated. We used cross-sectional Canadian Longitudinal Study on Aging data (n=27,765), collected between 2015 and 2018, to explore the potential confounding or modifying effects of personality traits on the associations between social isolation and loneliness score among 46-90 year-olds. We estimated associations using ordered logistic regression models, whereby the outcome was the loneliness score and exposures were social isolation and each of five personality traits (extraversion, agreeableness, conscientiousness, emotional stability, and openness). We produced three sets of models (social isolation only; personality trait only; and social isolation, personality trait and their interaction) and conducted all analyses stratified by sex and adjusting for potential confounders. We found that, for both males and females, social isolation and each of the five personality traits were associated with loneliness scores (p< 0.001), however, with only one exception (openness among females), there was no statistical evidence that personality modified the association between social isolation and loneliness. Our findings have potential implications for research assessing the health effects of social connection and personality as well as intervention studies that target social isolation as means of addressing loneliness.

